# Evaluation of additive manufacturing processes in the production of oculo-palpebral prosthesis

**DOI:** 10.12688/f1000research.111231.1

**Published:** 2022-05-09

**Authors:** Diego Eyzaguirre, Rodrigo Salazar-Gamarra, Salvatore Binasco Lengua, Luciano Lauria Dib

**Affiliations:** 1 University of Engineering and Technology - UTEC, Lima, Peru; 2 Plus Identity Institute, Sao Paulo, Brazil; 3 Norbert Wiener University - Digital Transformation Research Center, Lima, Peru

**Keywords:** Maxillofacial prosthesis, anaplastology, Digital Models, Additive Manufacturing, 3D-Printing

## Abstract

**Background:** Prosthetic restorations are made to adapt or attach missing human parts in order to restore function and appearance. Maxillofacial defects connote a greater impact on patients, since the face cannot be concealed, and all the senses of the human body are expressed in it. Therefore, in order to restore the patient’s quality of life, they are the ones that require the best possible adaptation to the characteristics of the patients.

**Methods:** For the maxillofacial prostheses to fit patients, they must be personalized for each patient. The NGO “Mais Identidade” is a multidisciplinary team that specializes in the rehabilitation of patients with maxillofacial trauma. They use digital manufacturing as a tool to manufacture personalized maxillofacial prostheses for patients. With the help of the NGO, the following research is conducted with the purpose of evaluating different methods of additive manufacturing, 3D printing, in order to select the equipment that suits the needs of the method used in the manufacture of maxillofacial prostheses. To this end, eyelid models will be manufactured in different additive manufacturing equipment, and these will be evaluated according to their economic, physical, and aesthetic characteristics.

## 1. Introduction

Prosthesis encompasses a wide range of mechanisms for the replacement of body parts, in order to rehabilitate the characteristics of the missing part in function or appearance. Within them, there is a branch known as “maxillofacial prostheses”, among which are nasal, ear, oculo-palpebral prostheses, *etc.* ^1^ They are generally used by patients who have suffered cancer (52%), deformities due to accidents (17%) and/or congenital diseases (19%), among others. ^2^

Most of the prostheses, being systems that simulate missing body parts, must be personalized for each patient, which tends to raise their costs. Traditional manufacturing methods require a lot of professional and patient time. In addition, in the public health system, when the service exists and is covered, there are patients who wait between 6 months and 2 years for their rehabilitations. Once the appointment is obtained, they can only be served between 30 minutes to 2 hours per appointment due to the shortage of time and high demand. With the method proposed by the NGO “Mais Identidade”, global work times can be reduced by half. ^3^

With the aim of optimizing the process and making prostheses more accessible and of good quality, ^4^ both data collection and manufacturing, a digital manufacturing process is chosen. Which consists of: data collection, such as digitization through 3D scanning or photogrammetry; prosthesis design (reverse engineering software) and rapid prototyping (3D printing). ^5^^,^^6^

In the manufacturing process of these prostheses, the patient’s self-perception, emotional stability, personality characteristics and social circumstances are the most important factors when treating maxillofacial defects, as well as the rehabilitation process. ^7^^,^^8^

In this sense, the purpose of this research is to compare additive manufacturing mechanisms, from a 3D model of the oculo-palpebral model for a future maxillofacial prosthesis, obtained from the “Mais Identidade” Method. It will be evaluated which of the additive manufacturing mechanisms achieves the best reproduction of the leather details and maintains the desired dimensional properties, according to the original file. To select the manufacturing method, the oculo-palpebral models manufactured in 7 different 3D printing mechanisms were evaluated, according to their economic, physical and aesthetic characteristics.

The structure of this paper is the following: section 2 briefly details the printing techniques used for the testing; section 3 describes the procedure of the experiments; section 4 presents and evaluates the results; section 5 discusses the evaluations and the context in which they were made; section 6 proposes suggestions for future work and research conclusions.

## 2. Resin 3D printing comparison

It is the process by which physical objects are generated from 3D digital files. These files, prior to being printed, go through software in which they are divided into thin layers, with the desired printing characteristics (speed, layer thickness, *etc.*). ^9^ For the “Mais Identidade” method, layer levels in the range of 100 μ to 16 μ are used to obtain a high level of detail.

### 2.1 Fused Deposition Modelling (FDM)

It is the most used method of 3D printing. This method uses a thermoplastic filament that goes through a heating system where it is heated to a temperature at which it is moldable and extruded to take the desired shape. ^10^ For the present investigation, PLA filament will be used as a material for printing the prototypes due to its printing practicality, low cost and because it is the most used material in this technology.

### 2.2 Stereolithography

This method covers liquid resins that go through a photopolymerization process, which exposes them to a specific range of light, with which they undergo a chemical reaction that solidifies them. ^10^ This technology is usually faster and has a higher level of finish than FDM. Within stereolithography, there are variants such as ^11^:

(1) SLA: A UV laser cures the resin point by point in the resin tank using a projector and a set of mirrors.
(2) LCD: An LCD screen projects the UV light and passes through a filter that allows the exposure of light in the necessary points.
(3) DLP: A projector emits light and through a mirror generates the shapes to be printed layer by layer.

The resins used, both in SLA and LCD and DLP, go through the photopolymerization process when interacting with a light range of 405 nm. Generally, each equipment uses its own resin, so basic light curing resins will be used for prototypes.

### 2.3 POLYJET

This method is based on the injection of polymers that are cured by ultraviolet light. This technology stands out for its high speed and high print resolution, as well as being able to reproduce functional models without the need to assemble them. ^12^ For this research, Vero black was used to print the model, and Sup706 was used for the support.

## 3. Methods

**Figure 1.**
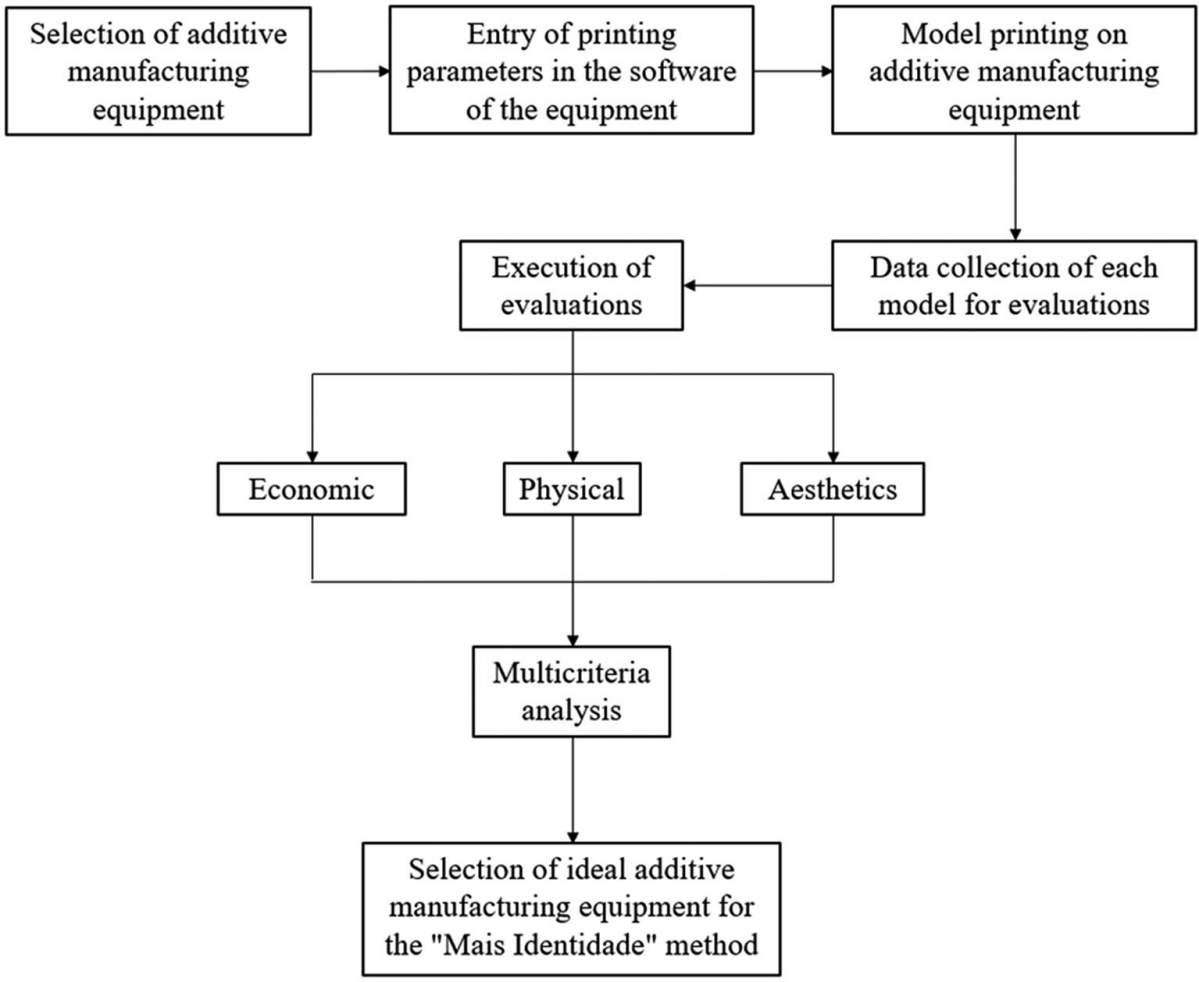
Methodological flowchart.

The selection of the ideal additive manufacturing equipment for printing models that are used in the manufacturing process of maxillofacial prostheses by the “Mais Identidade” method is detailed in Figure 1.

### 3.1 Selection of additive manufacturing equipment

The following technologies were used for the tests: FDM, SLA, LCD, DLP and POLYJET. Table 1 specifies the characteristics of the equipment selected for its good resolution and precision.

**Table 1.** Characteristics of additive manufacturing equipment.

Technology	Equipment	Characteristics		
		Print speed	Layer thickness	Resolution
FDM	Mini L	0-120 mm/s	100 μm	100 μm
LCD	Photon S	20 mm/hr	20 μm	47.5 μm
	DS-200	20 mm/hr	25 μm	75 μm
	Phrozen Shuffle XL	20 mm/hr	25 μm	75 μm
DLP	MoonRay S	3.81–25.4 mm/hr	20 μm	100 μm
	PRO95	12.7–50.8 mm/hr	20 μm	95 μm
POLYJET	Objet500 Connex3	-	16 μm	600 dpi

### 3.2 Entry of printing parameters in the software of the equipment

For the tests, the model to be printed was a clinical case of a 75-year-old patient with an oculo-palpebral trauma. For the input parameters, the best printing options were selected: per layer, material to be used, printing temperature (FDM), exposure times (SLA, LCD, DLP), *etc.*

### 3.3 Model printing on additive manufacturing equipment

For the tests, three impressions per equipment were made, to make an average with the data, based on the Design of Experiments theory. The printed models, as they require supports, must go through a post process that eliminates them to obtain the final model.

### 3.4 Data collection of each model for evaluations

For each impression, the data of the printing parameters were taken, to register them and carry out the economic and physical evaluations.

3.4.1 Economic data collection

To carry out the economic evaluations of each equipment, the data shown in Table 2, must be collected from each impression.

**Table 2.** Printing data by model.

Cost per liter of resin (Clk)	
Print volume (V)	
Printing time (T)	
Equipment Value (EV)	
Rescue Value (RV)	
Useful life (UL)	
Days per year (Ds)	365 days/year
hours per day (Hr)	24 hr/day
Design (D)	0 hr
Specifications (E)	560/hr
Control (C)	T*1%
Post Printing (PP)	2060/hr
Man Hour (MH)	15 $/hr

Using the previous data, the cost was calculated using the following equations:

1. Cost of material used
$$\mathrm{MC}=\frac{C*V}{1000}$$
  Where:
  •
$\mathrm{MC}:\text{Material Cost}\;\left(\$\right)$

  •
$C:\text{Cost}\;\mathrm{per}\;\text{liter or kilogram}\;\left(S/\text{liter or kilogram}\right)$

  •
$V:\text{Print Volume}\;\left(\mathrm{mL}\;o\;\mathrm{gr}\right)$

2. Cost hours machine worked
MHC=T∗MH
  Where:
  •
$\mathrm{MHC}:\text{Machine Hour Cost}\;\left(\$\right)$

  •
$T:\text{Printing Time}\;\left(\mathrm{Hr}\right)$

  •
$\mathrm{MH}:\text{Machine Hours}\;\left(\$/\mathrm{hr}\right)$

  Taking into consideration that the Machine Hour is calculated as follows:
$$\mathrm{MH}=\frac{\mathrm{EV}-\mathrm{RV}}{\mathrm{UL}*D*\mathrm{Hr}}$$
  Where:
  •
$\mathrm{EV}:\text{Equipment Value}\;\left(\$\right)$

  •
$\mathrm{RV}:\text{Rescue Value}\;\left(\$\right)$

  •
$\mathrm{UL}:\text{Useful Life}\;\left(\text{years}\right)$

  •
$D:\text{Days}\;\mathrm{per}\;\text{Year}\;\left(\text{days}/\text{year}\right)$

  •
$\mathrm{Hr}:\text{Hours}\;\mathrm{per}\;\mathrm{day}\;\left(\text{hours}/\mathrm{day}\right)$

3. Cost man-hour worked
$$\mathrm{CMH}=\left(D+E+C+\mathrm{PP}\right)*\mathrm{MH}$$
  Where:
  •
$\mathrm{CMH}:\text{Cost}\;\mathrm{per}\;\mathrm{Man}\;\text{Hour}\;\left(\$\right)$

  •
$D:\text{Design}\;\left(\mathrm{Hr}\right)$

  •
$E:\text{Especifications}\;\left(\mathrm{Hr}\right)$

  •
$C:\text{Control}\left(\mathrm{Hr}\right)$

  •
$\mathrm{PP}:\text{Post Printing}\;\left(\mathrm{Hr}\right)$

  •
$\mathrm{MH}:\mathrm{Man}\;\text{Hour}\;\left(\$/\mathrm{hr}\right)$

  • Taking into consideration that the cost of Man Hour is a fixed cost of $15/hr
4. Printing cost
PC=MC+MHC+CMH

3.4.2 Physical data collection

**Figure 2.**
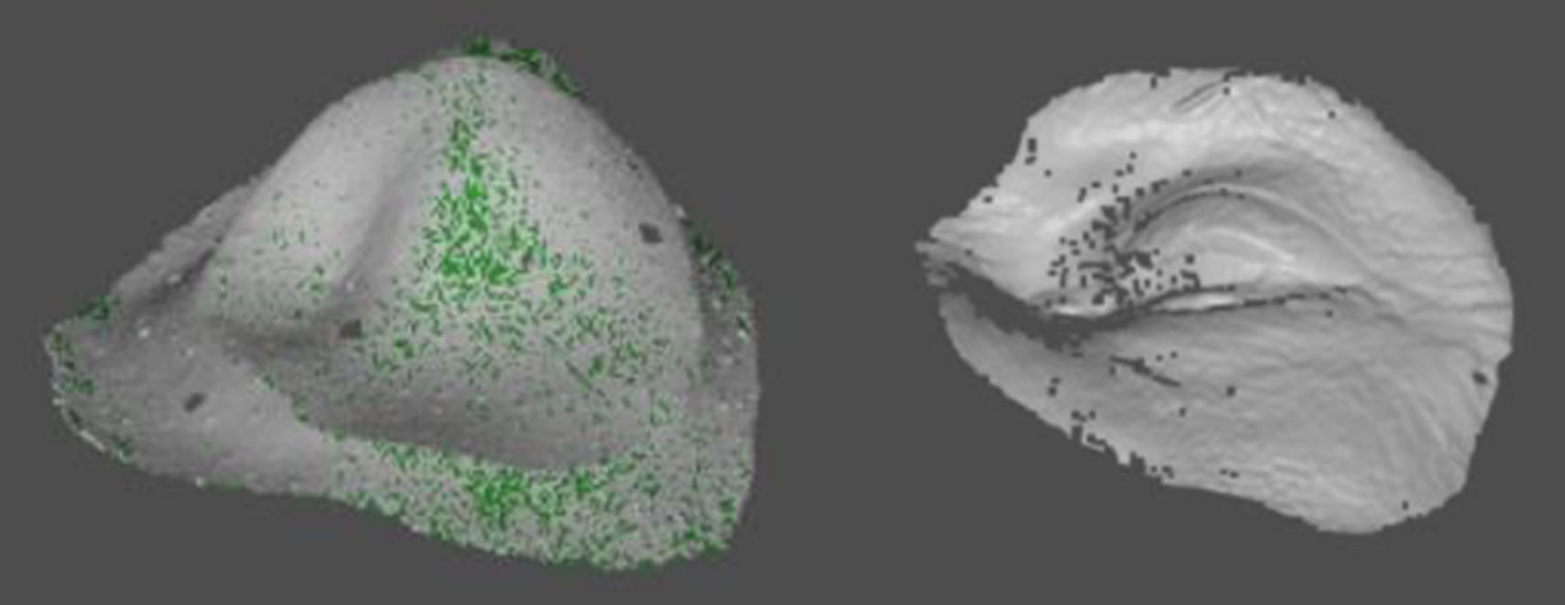
Front and back face scan.

For physical evaluations, each printed model is digitized and compared to the original digital file. To do this, each model is covered with developer spray to obtain better scans; reference points are placed on the back face of the model; in the ZEISS software it is scanned with the COMET 8M model; the rotary option is selected, with 10 stops and the back and front of the model are scanned, obtaining the results shown in Figure 2.

**Figure 3.**
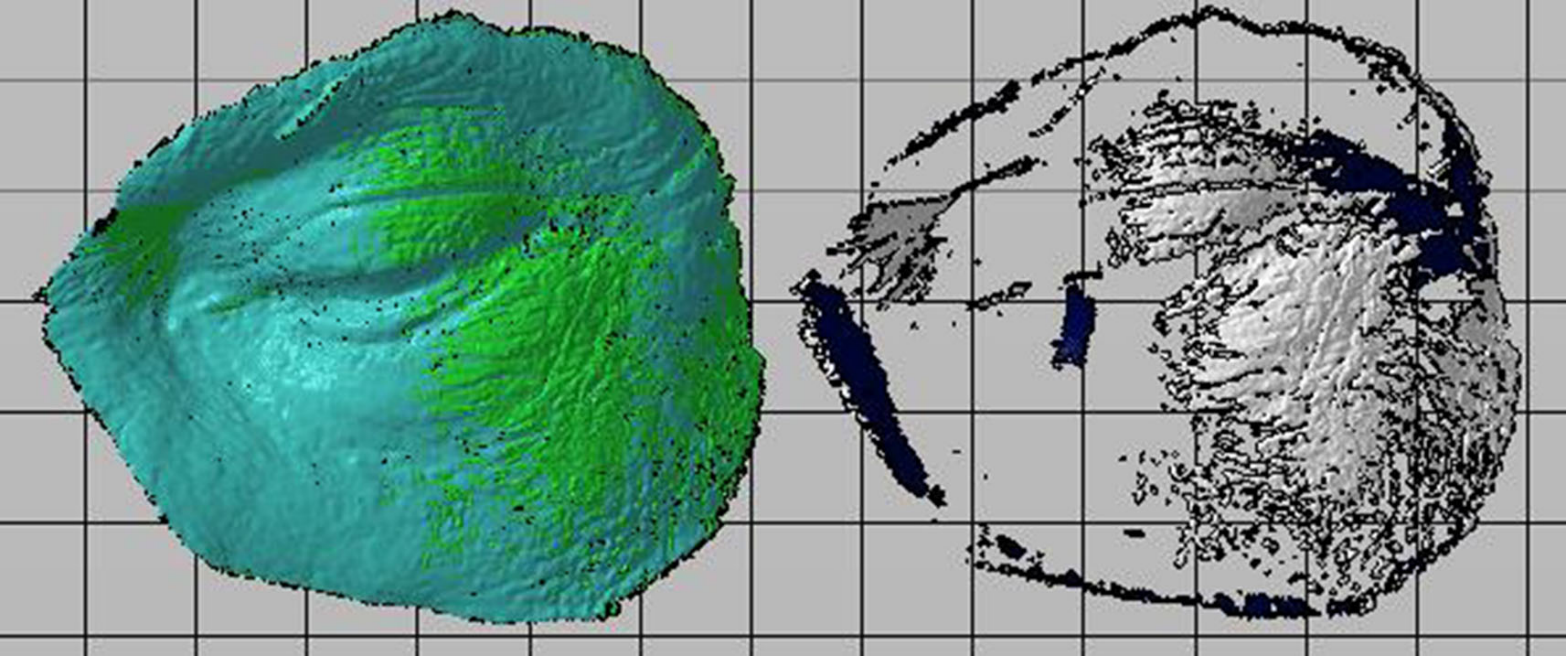
Alignment and boolean of digitized and original models.

With help of the reference points, the two shots are coupled to turn it into a single digitized model. Finally, the digitized model is compared with the original digital model and the deviation between both is obtained for each case, as shown in Figure 3.

### 3.5 Execution of evaluations

After registering the necessary data, economic, physical and aesthetic evaluations are made. Each evaluation has a score from 1 to 5, where 5 (five) is the model with the best features and 1 (one) the one with the lowest.

In the economic evaluation, the manufacturing cost for each prototype was considered for the score. In the physical evaluation, the precision was obtained according to the mean deviation for the models printed by each device. The aesthetic evaluation was carried out by Dr. Rodrigo Salazar, a specialist in the maxillofacial rehabilitation, according to his appreciation of the prototypes.

### 3.6 Multicriteria analysis

After evaluating the prototypes by the established parameters, the final scores were multiplied equally, 33.33% per evaluation. Once the scores were multiplied by the weights of each factor, the totals were added.

## 4. Results

### 4.1 Oculo-palpebral model printing

4.1.1 Mini L

**Figure 4.**
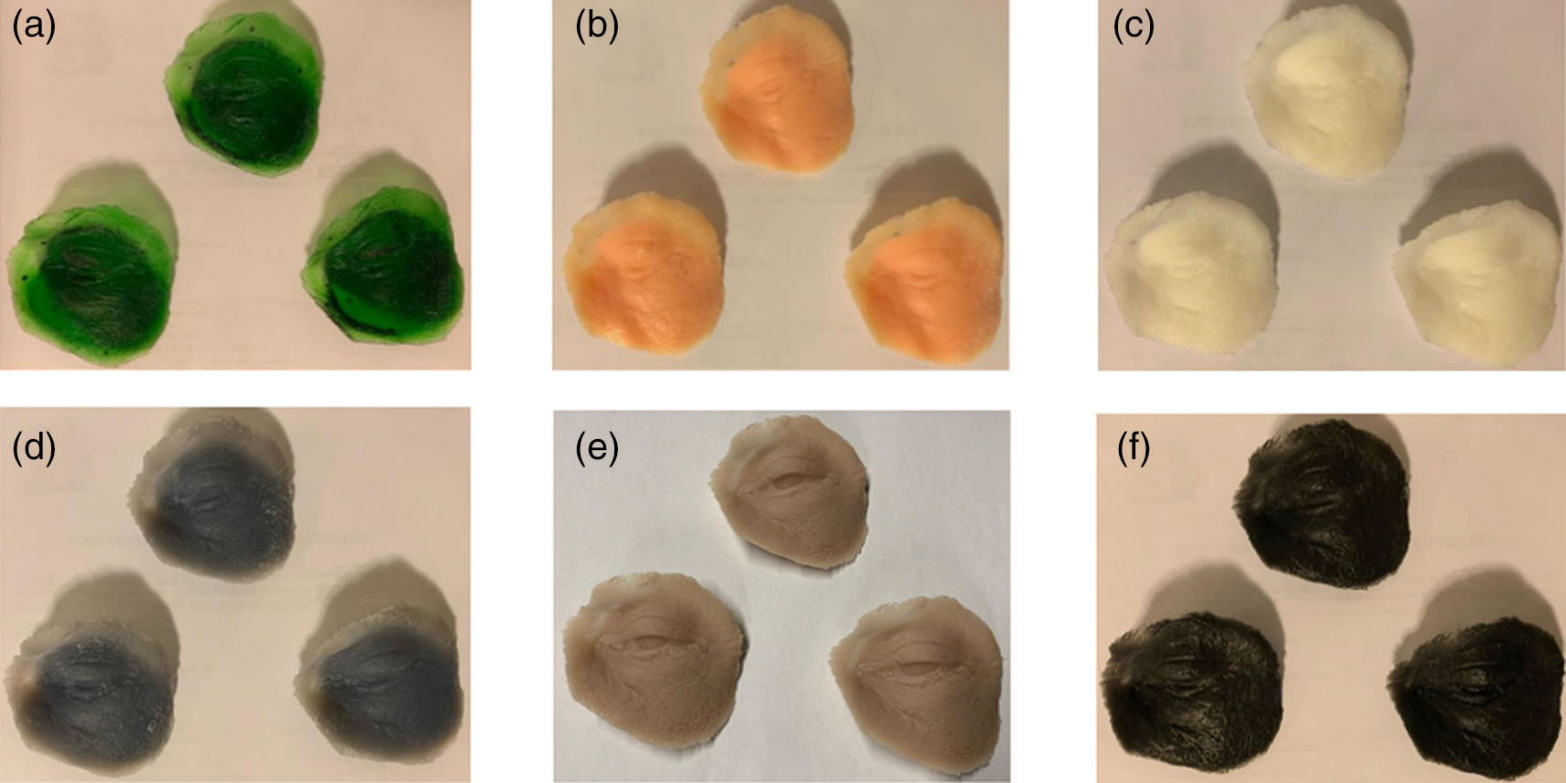
Oculo-palpebral model printed in: (a) Photon S, (b) DS-200, (c) Phrozen Shuffle XL, (d) Moon Ray S, (e) PRO 95 and (f) Objet500 Connex3.

For printing on the MINI L, with FDM technology, the 3DTALK slicer was used and parameters are shown in Table 3. The model is shown in Figure 4.

**Table 3.** Printing parameters for Mini L.

Filament	Layer thickness	Wall thickness	Print speed	Fill density	Support density
White PLA	0.1 mm	0.8 mm	40 mm/s	15%	15%

The model was not reproduced correctly, since it has complex parts that cannot be reproduced by FDM printers. For this reason, it was decided to leave aside the FDM technology in the evaluation.

4.1.2 Photon S

For printing on the Photon S, with LCD technology, the CHITUBOX slicer was used and parameters are shown in Table 4. The model is shown in Figure 4.

**Table 4.** Printing parameters for Photon S.

Resin	Layer thickness	Base layers	Base exposure	Normal exposure	Support density
Basic	0.02 mm	3	50 s	8 s	15%

4.1.3 DS-200

For printing on the DS-200, with LCD technology, the 3DTALK slicer was used and parameters are shown in Table 5. The model is shown in Figure 4.

**Table 5.** Printing parameters for DS-200.

Resin	Layer thickness	Base layers	Base exposure	Normal exposure	Support density
Modelo	0.025 mm	3	30 s	4 s	15%

4.1.4 Phrozen Shuffle XL

For printing on the Phrozen Shuffle XL, with LCD technology, the CHITUBOX slicer was used and parameters are shown in Table 6. The model is shown in Figure 4.

**Table 6.** Printing parameters for Phrozen Shuffle XL.

Resin	Layer thickness	Base layers	Base exposure	Normal exposure	Support density
Modelo	0.025 mm	3	30 s	4 s	15%

4.1.5 MoonRay S

For printing on the MoonRay S, with DLP technology, the RayWare slicer was used and parameters are shown in Table 7. The model is shown in Figure 4.

**Table 7.** Printing parameters for MoonRay S.

Resin	Layer thickness	Support thickness	Support force
Model Gray	0.02 mm	Low	Low

4.1.6 PRO95

For printing on the PRO95, with DLP technology, the RayWare slicer was used and parameters are shown in Table 8. The model is shown in Figure 4.

**Table 8.** Printing parameters for PRO95.

Resin	Layer thickness	Support density	Support force
Die & Model Tan	0.05 mm	Low	Low

4.1.7 Objet500 Connex3

For printing on the Objet500 Connex3, with Polyjet technology, a proprietary slicer was used the parameters are shown in Table 9. The model is shown in Figure 4.

**Table 9.** Printing parameters for Objet500 Connex3.

Materials	Layer thickness	Support density	Support force
Varoblack y Sup706	0.016 mm	Low	Low

### 4.2 Model data collection

The printing data per piece are shown in Table 10. Values such as: Days per year (Ds), Hours per day (Hr), Design, Specifications, Control and Post Printing Man-Hours are specified in Section 3.4.1.

**Table 10.** Printing data per model.

	Photon S	DS-200	Phozen XL	MoonRay S	PRO95	Objet500 Connex3
Cost per liter of resin (Clk)	90 $/L	180 $/L	180 $/L	200 $/L	200 $/L	201 $/Kg
Print volume (V)	15 mL	29 mL	20 mL	25 mL	28 mL	77 gr
Printing time (T)	6.33 hr	4.75 hr	5.8 hr	5.46 hr	1.68 hr	1.5 hr
Equipment Value (EV)	899 $	6000 $	2599 $	6500 $	11000 $	330000 $
Rescue Value (RV)	89 $	600 $	250 $	650 $	1100 $	33000 $
Useful life (UL)	0.5 years	2 years	1 years	5 years	6 years	6 years

Using the data obtained and equation 3.1 – 3.5, the printing cost was obtained as shown in Table 11.

**Table 11.** Model printing cost.

	Photon S	DS-200	Phozen XL	MoonRay S	PRO95	Objet500 Connex3
Material cost	$ 1.35	$ 5.27	$ 3.60	$ 5.00	$ 5.60	$ 15.50
Machine hour cost	$ 1.17	$ 1.46	$ 1.56	$ 0.73	$ 0.32	$ 8.47
Cost per man hour	$ 7.20	$ 6.96	$ 7.12	$ 7.07	$ 6.50	$ 8.98
Printing cost	$ 9.72	$ 14.70	$ 12.27	$ 12.80	$ 12.42	$ 32.95

**Figure 5.**
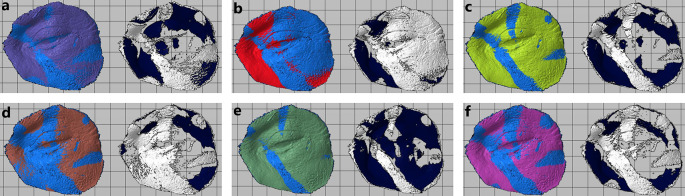
Scanned oculo-palpebral model of: (a) Photon S, (b) DS-200, (c) Phrozen Shuffle XL, (d) Moon Ray S, (e) PRO 95 and (f) Objet500 Connex3.

As a result of the alignment and boolean cut of the digitized models with the originals, as shown in Figure 5, the deviation between them and the corresponding physical data in Table 12 is obtained.

**Table 12.** Deviation between original and printed models.

	Photon S	DS-200	Phozen XL	MoonRay S	PRO95	Objet500 Connex3
Volume of model 1	10.974	10.435	11.553	10.599	10.873	11.090
Volume of Boolean 1	0.131	0.487	0.174	0.244	0.168	0.477
Deviation 1	1.350%	5.018%	1.793%	2.514%	1.731%	4.914%
Volume of model 2	11.163	10.436	11.454	10.664	10.84	11.220
Volume of Boolean 2	0.149	0.544	0.166	0.354	0.156	0.519
Deviation 2	1.535%	5.605%	1.710%	3.647%	1.607%	5.347%
Volume of model 3	11.227	10.335	11.497	10.613	10.828	11.209
Volume of Boolean 3	0.128	0.656	0.159	0.235	0.173	0.484
Deviation 3	1.319%	6.759%	1.638%	2.421%	1.782%	4.987%
Mean deviation	1.401%	5.794%	1.714%	2.861%	1.707%	5.083%

### 4.3 Evaluation of the models

4.3.1 Economic evaluation

As shown in Table 13, the equipment with the best score and the lowest printing cost is the Photon S. While the equipment with the lowest score and the highest printing cost is the Objet500 Connex3.

**Table 13.** Economic evaluation.

Printing cost range ($)	41 – 50	31 – 40	21 – 30	11 – 20	0 – 10
Score	1	2	3	4	5
Photon S					$ 9.72
DS-200				$ 13.70	
Phrozen Shuffle XL				$ 12.27	
MoonRay S				$ 12.80	
PRO95				$ 12.42	
Objet500 Connex3		$ 39.95			

4.3.2 Physical evaluation

As shown in Table 14, the devices that are in the range of deviation with the best score are: Photon S, Phrozen Shuffle XL and PRO95, while the devices with the highest deviation are: DS-200 and Objet500 Connex3.

**Table 14.** Physical evaluation.

Deviation range	8-10%	6-8%	4-6%	2-4%	0-2%
Score	1	2	3	4	5
Photon S					1.401%
DS-200			5.794%		
Phrozen Shuffle XL					1.714%
MoonRay S				2.861%	
PRO95					1.707%
Objet500 Connex3			5.083%		

4.3.3 Aesthetic evaluation

As shown in Table 15, the prints of the PRO95 and Objet500 Connex 3 obtained the best score, while the equipment with the least score was the DS-200 printer.

**Table 15.** Aesthetic evaluation.

Deviation range	Low	Regular low	Regular	Regular good	Good
Score	1	2	3	4	5
Photon S				X	
DS-200		X			
Phrozen Shuffle XL			X		
MoonRay S				X	
PRO95					X
Objet500 Connex3					X

4.3.4 Multicriteria evaluation

Finally, as shown in Table 16, the equipment with the best combined score were the Photon S and the PRO95, with 4.67 out of 5.

**Table 16.** Multicriteria evaluation.

Equipment	Physical evaluation	Economic evaluation	Aesthetic evaluation	Total
Weight	33.33%	33.33%	33.33%	100.0%
Photon S	1.67	1.67	1.33	4.67
DS-200	1.00	1.33	0.67	3.00
Phrozen XL	1.67	1.33	1.00	4.33
MoonRay S	1.33	1.33	1.33	4.00
PRO95	1.67	1.33	1.67	4.67
Objet500 Connex3	1.00	0.67	1.67	3.33

The Photon S, due to its LCD technology, is an economical equipment, with a high level of precision and suitable for using a wide range of resins. The disadvantages of the equipment are its printing volume, 11.5×6.5×16.5 cm, in addition to having components with a short useful life, LCD screen, FEP film, *etc.*

The PRO95, due to its DLP technology, has an excellent level of precision, its large printing volume, as well as being one of the fastest stereolithography equipment on the market. The disadvantages of the equipment are its high cost, which exceeds $10,000 and its manufacturing cost is almost four times that of the Photon S.

For the choice of equipment to be used in the “Mais Identidade” methodology, the volume of work, acquisition capacity, reliability of the equipment, among other parameters, must be considered.

## 5. Discussion

In the investigation, a standardized methodology was proposed in order to minimize the variation between tests. As in the printing process in LCD and DLP equipment, both in the steps in the use of the slicer and in post printing. In the same way, this must be done in the digitization process with the Comet 8M scanner, since it has labor requirements for optimal results.

Previous research on additive manufacturing (3D printing) demonstrates the feasibility of its use for manufacturing processes of maxillofacial prostheses. Unlike these investigations, this one focuses on the equipment within the Peruvian market for the selection of the ideal team for the “Mais Identidade” process, evaluated based on the investigation of the Mais Identidade Institute, in conjunction with Dr. Rodrigo Salazar. Likewise, it was decided to use these seven pieces of equipment in the investigation due to time limits and accessibility to them.

## 6. Conclusions and future work

In the economic evaluation the Photon S printer obtained the best score with 9.72 dollars, unlike the Objet500 Connex3 which obtained the lowest score with 32.95 dollars. In the physical evaluation, the Photon S, Phrozen Shuffle XL and PRO95 obtained the best score, within the deviation range of 0–2%. While the DS-200 and Objet500 Connex3 equipment obtained the lowest score, within the deviation range of 4–6%. In the aesthetic evaluation, the doctor gave his appreciation for the printed models, highlighting those printed on the PRO95 equipment, while the equipment with the lowest results were the DS-200 and Objet500 Connex3.

When obtaining the final results, there are two teams with equal scores in the Multicriteria Analysis (Photon S and PRO95), both scored 4.67 points out of 5. The characteristics of each team should be taken into consideration: printing volume, printing speed, volume of work; as well as organizational factors: budget, conditions or workflow.

In this way, it is recommended in future research to broaden the spectrum of evaluation with criteria such as: printing capacity of each equipment, reliability of the equipment, volume of work, *etc.* As well as expanding the range of technologies and equipment to be evaluated, since with technological advances, equipment may arise that is more suited to the needs of each organization.
