# Supplementary material for: Evaluation of additive manufacturing processes in the production of oculo-palpebral prosthesis
Source: F1000Res. 2023 Feb 14;11:505. Originally published 2022 May 9. [Version 2] doi: 10.12688/f1000research.111231.2 (PMC10799225; doi:10.12688/f1000research.111231.2)
Supplement: Peer review history

# Reviewer report: Reviewer response for version 2

**DOI:** 10.5256/f1000research.144046.r234699

**Published:** 2024-01-19

**Authors:** Harish Kumar Banga

**Affiliations:** 1 National Institute of Fashion Technology, New Delhi, Delhi, India

The introduction should briefly describe what was previously published in relation to the topic under study, what was needed to carry out the research, the hypothesis and the purpose of the work.

 Review the introductory part of the work providing the information necessary for the reader to understand the problem under study.

**MATERIAL AND METHODS**

 Materials and methods section  is well elaborated, with a clear description of the scientific methodology applied in the study and a valid iconography that allows the reader an immediate understanding.

**RESULT ANALYSIS AND DISCUSSION**

 This part of the paper should be reorganized because it’s too confusing and hard to understand for the reader. The results should be reported in a separate section from the discussion.The introduction should briefly describe what was previously published in relation to the topic under study, what was needed to carry out the research, the hypothesis and the purpose of the work.

**References.**

 Some references are quite old . If possible, please switch with some more modern research. Some recent studies have been suggested in the reference section.

Is the work clearly and accurately presented and does it cite the current literature?

No

If applicable, is the statistical analysis and its interpretation appropriate?

Partly

Are all the source data underlying the results available to ensure full reproducibility?

Partly

Is the study design appropriate and is the work technically sound?

Partly

Are the conclusions drawn adequately supported by the results?

Partly

Are sufficient details of methods and analysis provided to allow replication by others?

Yes

Reviewer Expertise:

Additive Manufacturing, Ergonomics, CAD/CAM

I confirm that I have read this submission and believe that I have an appropriate level of expertise to confirm that it is of an acceptable scientific standard, however I have significant reservations, as outlined above.

## References

[1] : Reverse engineering and CAD/CAM application in the design of maxillofacial prosthesis. *International Journal on Interactive Design and Manufacturing (IJIDeM)* .2023; 10.1007/s12008-023-01503-5 10.1007/s12008-023-01503-5

# Reviewer report: Reviewer response for version 2

**DOI:** 10.5256/f1000research.144046.r163326

**Published:** 2023-02-21

**Authors:** Dinesh Rokaya

**Affiliations:** 1 Department of Clinical Dentistry, Walailak University International College of Dentistry, Walailak University, Bangkok, Thailand

The authors have made the necessary corrections and improved the manuscript.

 Figure 1 fitting the manuscript means Figure 1 should fit within the manuscript layout.

 The manuscript looks good and it can be approved.

Is the work clearly and accurately presented and does it cite the current literature?

Partly

If applicable, is the statistical analysis and its interpretation appropriate?

Partly

Are all the source data underlying the results available to ensure full reproducibility?

Yes

Is the study design appropriate and is the work technically sound?

Yes

Are the conclusions drawn adequately supported by the results?

Partly

Are sufficient details of methods and analysis provided to allow replication by others?

Partly

Reviewer Expertise:

NA

I confirm that I have read this submission and believe that I have an appropriate level of expertise to confirm that it is of an acceptable scientific standard.

# Reviewer report: Reviewer response for version 1

**DOI:** 10.5256/f1000research.122926.r150240

**Published:** 2022-10-03

**Authors:** Rodrigo de Faria Valle Dornelles

**Affiliations:** 1 Hospital Beneficência Portuguesa de São Paulo, São Paulo, Brazil; 2 Faculdade de Medicina Campus Guarujá, UNAERP, Guarujá, Brazil

The present study proposes to quantitatively evaluate the quality and cost results of different methods of producing oculo-palpebral prostheses by means of 3D printing. The authors have background and experience for the production of the piece and summarize the workflow. The additive manufacturing methods used and the calculation methods used to define costs are described. Quality was assessed objectively by Boolean principles and subjectively by one of the authors. The conclusion was based on findings with the formulas described and on a subjective assessment of the most appropriate method.

 Is the study design appropriate and is the work technically sound?

- The subjective assessment would be more appropriate if it were performed by more than one professional not connected to the study

Are sufficient details of methods and analysis provided to allow replication by others?

- It was not clear the parameters used for printing, especially with regard to FDM

Are all the source data underlying the results available to ensure full reproducibility?

- Same reason as before, and not clear why the FDM method was discarded

Is the work clearly and accurately presented and does it cite the current literature?

Yes

If applicable, is the statistical analysis and its interpretation appropriate?

I cannot comment. A qualified statistician is required.

Are all the source data underlying the results available to ensure full reproducibility?

Partly

Is the study design appropriate and is the work technically sound?

Partly

Are the conclusions drawn adequately supported by the results?

Yes

Are sufficient details of methods and analysis provided to allow replication by others?

No

Reviewer Expertise:

Plastic Surgery; Cranio-Maxillo-Facial Surgery; 3D researcher

I confirm that I have read this submission and believe that I have an appropriate level of expertise to confirm that it is of an acceptable scientific standard, however I have significant reservations, as outlined above.

# Response

**Published:** 2023-02-05

**Authors:** Salvatore Binasco Lengua

**Affiliations:** Plus Identity Institute, Peru

Thank you for the feedback.

 In a new version of the manuscript we have clarified the points you mentioned about printing parameters and ruling out FDM technology. We will also take into account the comment about an evaluator not linked to the study for future work.

# Reviewer report: Reviewer response for version 1

**DOI:** 10.5256/f1000research.122926.r150243

**Published:** 2022-09-23

**Authors:** Dinesh Rokaya

**Affiliations:** 1 Department of Clinical Dentistry, Walailak University International College of Dentistry, Walailak University, Bangkok, Thailand

This is an interesting study but needs significant corrections and editing of the manuscript.

**Abstract:**

- “Prosthetic restorations are made to adapt or attach missing human parts in order to restore function and appearance.” Please remove this line as this is generally for intraoral prostheses. And add one line on the role of the maxillofacial prosthesis in the restoration of the maxillofacial defect.
- Add objectives of this research.
- It is better to add some results.

**Introduction:**

- Please add some common biomaterials that can be printed for medical applications. ^1^
- In the introduction, it will be useful to add a paragraph on the ocular prosthesis in the restoration of the ocular defect. ^1^^,^^2^

**Materials and Methods:**

- Please add references for the data in Table 1, and Tables 4-9.
- Add references for the equations used.
- Please describe more on the statistical analysis.

**Results:**

- Figure 1 should be edited to make it better fitting in the manuscript.

**Discussion:**

- More discussion needs to be done comparing with other studies.
- Please add the limitations of this research.

**Conclusion:**

- It is long. Present the conclusion in a better way and make it concise.

**Overall:**

- Revision is required.
- Re-access is needed after the revision.

Is the work clearly and accurately presented and does it cite the current literature?

Partly

If applicable, is the statistical analysis and its interpretation appropriate?

Partly

Are all the source data underlying the results available to ensure full reproducibility?

Yes

Is the study design appropriate and is the work technically sound?

Yes

Are the conclusions drawn adequately supported by the results?

Partly

Are sufficient details of methods and analysis provided to allow replication by others?

Partly

Reviewer Expertise:

Dentistry, Prosthodontics, Maxillofacial Prosthetics, Dental Biomaterials

I confirm that I have read this submission and believe that I have an appropriate level of expertise to confirm that it is of an acceptable scientific standard, however I have significant reservations, as outlined above.

## References

[1] : 3D-Printed Biomaterials in Biomedical Application.2022; 10.1007/978-981-16-7152-4_12 319-339 10.1007/978-981-16-7152-4_12

[2] : Prosthetic rehabilitation of an ocular defect with post-enucleation socket syndrome: A case report. *Saudi Dent J* .2014;26(1) : 10.1016/j.sdentj.2013.12.006 29-32 10.1016/j.sdentj.2013.12.006 24526826 PMC3923168

[3] : Magnification of Iris through Clear Acrylic Resin in Ocular Prosthesis. *J Funct Biomater* .2022;13(1) : 10.3390/jfb13010029 10.3390/jfb13010029 35323229 PMC8952227

# Response

**Published:** 2023-02-05

**Authors:** Salvatore Binasco Lengua

**Affiliations:** Plus Identity Institute, Peru

Thank you for the feedback. A new version of the manuscript was uploaded.

 We did the changes in the abstract for a better preview of the paper and added the topics you recommended in the introduction. The Data in Table1 and 4-9 was made by us, based on the printing parameters on the printers used. We also correct the discussion and conclusion.

 Just a question:

 What exactly do you mean by Figure 1 best fitting the manuscript?
